# Innervation of an Ultrasound-Mediated PVDF-TrFE Scaffold for Skin-Tissue Engineering

**DOI:** 10.3390/biomimetics9010002

**Published:** 2023-12-20

**Authors:** Jennifer A. Westphal, Andrew E. Bryan, Maksym Krutko, Leyla Esfandiari, Stacey C. Schutte, Greg M. Harris

**Affiliations:** 1 Department of Biomedical Engineering, University of Cincinnati, Cincinnati, OH 45221, USA; westphja@mail.uc.edu (J.A.W.); krutkomm@mail.uc.edu (M.K.); esfandla@ucmail.uc.edu (L.E.); schuttsy@ucmail.uc.edu (S.C.S.); 2 Department of Chemical and Environmental Engineering, University of Cincinnati, Cincinnati, OH 45221, USA; bryanae@mail.uc.edu; 3 Department of Environmental and Public Health Sciences, University of Cincinnati, Cincinnati, OH 45267, USA; 4 Department of Electrical and Computer Science, University of Cincinnati, Cincinnati, OH 45221, USA; 5 Neuroscience Graduate Program, University of Cincinnati College of Medicine, Cincinnati, OH 45221, USA

**Keywords:** tissue-engineered skin, PVDF-TrFE, biomaterials, peripheral nerve, ultrasound, extracellular matrix

## Abstract

In this work, electrospun polyvinylidene-trifluoroethylene (PVDF-TrFE) was utilized for its biocompatibility, mechanics, and piezoelectric properties to promote Schwann cell (SC) elongation and sensory neuron (SN) extension. PVDF-TrFE electrospun scaffolds were characterized over a variety of electrospinning parameters (1, 2, and 3 h aligned and unaligned electrospun fibers) to determine ideal thickness, porosity, and tensile strength for use as an engineered skin tissue. PVDF-TrFE was electrically activated through mechanical deformation using low-intensity pulsed ultrasound (LIPUS) waves as a non-invasive means to trigger piezoelectric properties of the scaffold and deliver electric potential to cells. Using this therapeutic modality, neurite integration in tissue-engineered skin substitutes (TESSs) was quantified including neurite alignment, elongation, and vertical perforation into PVDF-TrFE scaffolds. Results show LIPUS stimulation promoted cell alignment on aligned scaffolds. Further, stimulation significantly increased SC elongation and SN extension separately and in coculture on aligned scaffolds but significantly decreased elongation and extension on unaligned scaffolds. This was also seen in cell perforation depth analysis into scaffolds which indicated LIPUS enhanced perforation of SCs, SNs, and cocultures on scaffolds. Taken together, this work demonstrates the immense potential for non-invasive electric stimulation of an in vitro tissue-engineered-skin model.

## 1. Introduction

Peripheral neuropathy in the skin presents diverse challenges in patients, ranging from moderate discomfort to life-long disabilities. Peripheral nervous system (PNS) injury is a result of traumatic damage such as compressing, stretching, or separating of the peripheral nerve and is the most persistent disabling neuromuscular impediment of second- and third-degree burns on the skin [1,2]. In cutaneous tissue, PNS damage leaves patients with discomfort, weakness, tingling or numbness, and sharp pain that can vary in severity and complexity. It is reported that as high as a 29% incidence of peripheral neuropathy occured in 66 burn patients [3], while a 9.73% prevalence in 370 burn patients was reported elsewhere [4]. Approximately 1.1 million people in the US suffer from burn injuries that require medical intervention leading to 4500 fatalities yearly with another 10,000 deaths attributed to burn-related infections occurring each year [5].

Medical treatments for major skin loss include autografts, allografts, xenografts, and more recently, tissue-engineered skin substitutes (TESSs) that are implemented in severe cases. However, conventional wound-healing therapies face numerous limitations that often leave patients with neuropathologic pain, itch, or loss of sensory function following large skin injuries. Donated skin is scarce and poses a risk of immune response, while autografts are inconvenient due to requiring multiple surgeries and comorbidities. Commercially available options such as TESSs vary in composition and have different restrictions upon them. As skin is the body’s largest organ, TESSs have advanced remarkably in recent years, and yet a comprehensive, functional, and innervated TESS remains elusive. Further, all TESSs have several limitations in common: a lack of innervation, weak mechanical properties, limited vasculature, and high costs [6]. Since engineered skin substitutes fail to reconstruct nerves and skin appendages including hair follicles, sweat glands, and sebaceous glands, an application of a TESS often results in little to no hair growth and sweat gland formation, as well as loss of skin pigmentation and sensation. This can negatively affect the quality of life of some patients [7]. Weak mechanics can cause TESSs to be easily damaged during fabrication or by in vivo tensile and shear forces following transplantation. Without extensive innervation, conventional TESSs fall short of restoring sensory function as well as reducing chronic pain or itch post transplantation. The literature has shown that graft reinnervation of patient’s native sensory neurons is not only crucial for regaining sensation, but also for the regeneration of damaged tissue [8,9,10]. As such, there is a high demand for new therapeutics to aid in nerve regeneration and integration of cutaneous tissue.

An understudied component of TESSs and neural integration is the Schwann cell (SC), which is the primary glial cell of the PNS. SCs produce myelin to ensheath axons and are important for normal function and maintenance of peripheral nerves. SCs support axonal outgrowth via the expression of many hormones and neurotrophins, especially after injury [11,12,13], to promote healthy axon growth and prevent early programmed neuronal apoptosis [14,15,16]. Additional studies show SCs express surface cell adhesion molecules and construct the basal lamina to condition axons for myelin formation [11,16,17,18]. As SCs play an integral role in the extracellular matrix (ECM), proper mechanics of the TESS are also needed. These mechanical properties of skin substitutes are vitally important as they define the wound-healing response by SCs and determine the restoration of native ECM and integration with surrounding tissue [19]. Therefore, optimizing the tensile properties of skin substitutes is crucial for functional wound recovery.

To address these needs, a polyvinylidene-trifluoroethylene (PVDF-TrFE) scaffold was selected for its biocompatibility and piezoelectric properties to promote Schwann cell (SC) elongation and sensory neuron (SN) extension without the need for electrodes, and for its mechanical strength. PVDF-TrFE also possesses tunable chemical, electrical, and physical properties that support a favorable cell-scaffold environment, making it an ideal platform for nerve regeneration and integration. PVDF-TrFE has also previously shown to promote axon regeneration through neurite extension both in vitro [20,21,22,23,24] and in vivo [25]. As it is piezoelectric, PVDF-TrFE is capable of electrical stimulation via mechanical deformations to stimulate cells and tissue. PVDF-TrFE electric potential can be activated using low-intensity pulsed ultrasound (LIPUS) as well as mechanical deformation through modalities such as negative pressure.

Recent work has also shown LIPUS can enhance neuronal activity by regulating secretion of cytokines and neurotrophic factors, decreasing inflammatory responses, and improving soft-tissue regeneration [26,27,28]. The literature also suggests LIPUS application to nerve injuries has a positive impact on promyelinating genes [29]. Apart from inducing PNS repair, LIPUS also expedites skin healing by approximately 40% through activation of Rac1 in both dermal and epidermal layers [30]. It has been shown that an inverse relationship between wound size and ultrasound intensity after skin grafting exists, indicating that ultrasound could be responsible for healthy tissue formation and re-epithelization [31]. A meta-analysis performed further supports that LIPUS facilitates early healing processes in venous stasis and diabetic foot-ulcer patients [32]. Altogether, the combination of piezoelectric PVDF-TrFE and LIPUS has potential for not only addressing PNS injuries, but also as a prospective therapy for cutaneous healing and skin-graft integration. However, even with numerous potential advantages of LIPUS on skin and nerve repair, the complete effectiveness of ultrasound stimulation in neurodegenerative diseases remains intricate and understudied. Therefore, the implementation of LIPUS in PNS regeneration is still deficient. Applying this therapeutic modality to PVDF-TrFE has potential for addressing the lack of nerve integration into skin substitutes through further investigation of neurite alignment, elongation, and vertical perforation, which has not been completed before.

**Figure 1:**
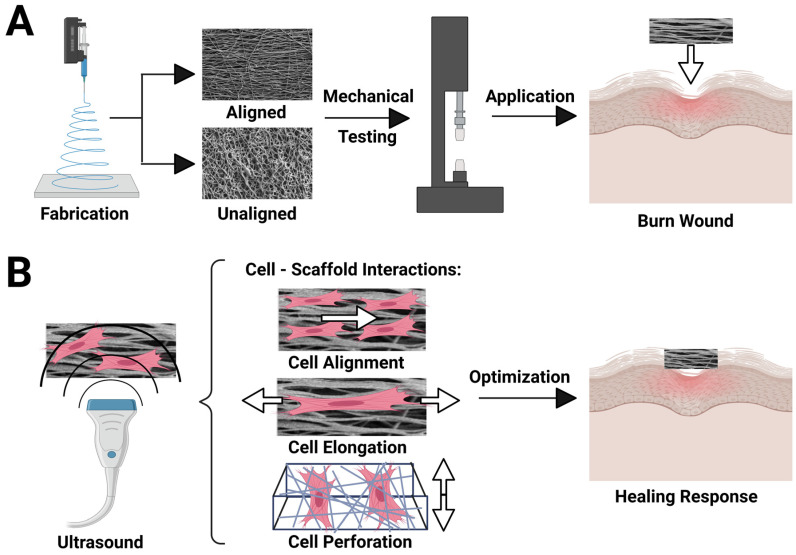
(**A**) Schematic displaying the experiments performed with aligned and unaligned PVDF-TrFE scaffolds. (**B**) Cell-scaffold interactions (alignment, elongation, and perforation) are analyzed before and after ultrasound stimulation to determine the most ideal parameters for skin-tissue engineering.

In this work, we explore the combinatorial effects of SCs in coculture with axonal outgrowth of SNs when exposed to LIPUS stimulation of piezoelectric PVDF-TrFE, aiming to determine how a piezoelectric scaffold and LIPUS increase neurite outreach for enhanced innervation. Material fabrication, tensile testing, and LIPUS stimulation for enhancing the desired cell-scaffold interactions in this novel work are outlined in Figure 1. Further, we have applied LIPUS to aligned and unaligned electrospun PVDF-TrFE scaffolds seeded with SCs and SNs to define the therapeutic parameters for maximum vertical neurite outreach in a model TESS. This work lays the foundation for future model TESSs using PVDF-TrFE as a modality for on-demand electrical control using LIPUS.

## 2. Materials and Methods

### 2.1. Fabrication of PVDF-TrFE Scaffolds

PVDF-TrFE scaffolds were prepared following our previous work [21,33,34]. Briefly, a polymer solution comprised 20% (*w*/*v*) PVDF-TrFE (70/30 mol) powder (PolyK Technologies) in a solvent of N,N-dimethylformamide (DMF):acetone 6:4 (*v*/*v*) was electrospun for 1, 2, or 3 h to fabricate approximately 10, 20, or 30 μm thick fiber mats, respectively. The polymer solution was poured into a 5 mL syringe with a 20-gauge needle, supplying a flow rate of 1 mL h^−1^. The needle tip was positioned 10 cm from the collector which was set at varying speeds to produce either aligned or unaligned fibers. Collection speeds of 200 RPM produced unaligned fibers, whereas 2000 RPM produced aligned fibers. The collector roll was covered by a conductive polymer liner (McMaster-Carr) onto which the PVDF-TrFE fibers were spun. A total of 18 kV was applied between the collector and the needle tip. Prior to cell culture, 1 × 1 cm squares of PVDF-TrFE were sterilized with 70% ethanol and incubated at 37 °C for 1 h followed by three PBS rinses. To improve hydrophilicity, scaffolds were incubated for 24 h in cell-specific media before seeding, detailed in Section 2.4 Cell Culture.

### 2.2. Porosity Measurement of PVDF-TrFE Fibers

Scaffold porosity was determined using an established method [23,35] for 1, 2, and 3 h spun aligned and unaligned scaffolds (*n* = 5). Briefly, the density of a sample scaffold (ρ_scaffold_) was calculated by measuring its dimensions and obtaining its mass. Porosity (%) was then determined with the following equation:

(1) $$Porosity\left(\%\right)=\left(1-\frac{\rho_{scaffold}}{\rho_{raw}}\right)\times 100\%$$

where the density of the raw PVDF-TrFE polymer powder (ρ_raw_) is 1.88 g cm^−3^ [21].

### 2.3. Mechanical Testing of PVDF-TrFE Fibers

For 1, 2, and 3 h, spun unaligned PVDF-TrFE scaffolds (*n* = 5) were tested, in tension, with a custom testing machine (100R6; TestResources, Shakopee, MN, USA). Scaffolds were stretched parallel to fiber direction until material rupture. Young’s modulus and ultimate tensile strength values were recorded for each scaffold thickness in MtestWR software to measure the tensile stiffness of PVDF-TrFE in response to the applied force. Scaffolds were cut into 2 × 2 cm squares and scaffold thicknesses were verified with electronic digital calipers (Absolute AOS Digimatic, Mitutoyo, Aurora, IL, USA) and an inverted Nikon A1R confocal microscope.

### 2.4. Cell Culture

RT-D6P2T rat Schwann cells (ATCC) were cultured in Dulbecco’s high-glucose-modified eagle’s medium (Cytiva) supplemented with 10% Bovine Calf Serum and 1% Pen/Step (Thermo Fisher, Waltham, MA, USA). SCs were routinely passaged upon reaching 80% confluence.

Primary adult SNs were purified from dorsal root ganglia (DRG) extracted from mice. DRG isolation surgeries were performed at the Davidson Lab at the University of Cincinnati’s medical campus in the Department of Anesthesia, following a protocol already established in the literature [36,37]. SNs were submerged in neurobasal-A medium supplemented with B-27 50× (Gibco) before being seeded on scaffolds.

Unmodified primary human keratinocytes (hKs) were obtained from human skin surgeries and purified [38]. hKs were cultured in MCDB 153 medium (Thermo Fisher, Grand Island, NY, USA) supplemented with human keratinocyte growth supplement (HKGS) and Pen/Strep.

Cells were seeded at 500 cells/mm^2^ (for scaffolds with SCs only), ¼ mE (mouse equivalent) (for scaffolds with SNs only), 250 SCs/mm^2^ with ¼ mE (for SC-SN cocultures), and 100 SC/mm^2^ with ¼ mE for ultrasound experiments. hKs for viability assays were seeded at 10 k cells/well in a 12-well plate (Thermo Fisher) for 9 days with fluorescence measurement every 48 h. All cultures were incubated at 37 °C, 95% relative humidity, and 5% CO_2_.

### 2.5. hK Viability Assay

hK viability on PVDF-TrFE was quantified using Presto-blue reagent (Thermo Fisher) using metabolic activity as a surrogate for viability [39]. hKs were seeded in a 12-well plate (control) or on PVDF-TrFE at 0 or 10 k cells/well, cultured for 9 days. The reagent-specific manufacturer’s protocol was followed (Thermo Fisher). Briefly, after seeding a 12-well plate with the above densities, 10 μL of Presto-blue and 90μL of medium and cells were pipetted into each well of a 96-well plate after culturing in a 12-well plate (500 μL of medium and cells with 55.5 μL of Presto-blue reagent added to each well). Presto-blue was added every 48 h and absorbance readings (relative fluorescence units) were determined via Cytation 5 Cell Imaging Multimode Reader (BioTek, Winooski, VT, USA), which were analyzed in Microsoft Excel. hK adhesion and proliferation were verified visually via immunofluorescence microscopy with DAPI at Day 1 and Day 9 of the assay.

### 2.6. Ultrasound Stimulation

Cells on PVDF-TrFE were stimulated with an ultrasound device (US PRO 2000™ 2nd Edition Model # DU3035) by placing the transducer underneath a 60 mm diameter polystyrene cell culture plate. SCs, SNs, and SC-SN cocultures seeded on 2 h aligned and 2 h unaligned PVDF-TrFE were divided into two groups: a stimulated group and a control group (no stimulation). The parameters of the ultrasound device included an output intensity of 0.08 W cm^−2^ ± 20% with a frequency of 1.0 MHz ± 10% as previously determined by the lab [34]. Scaffolds were stimulated for 5 min 24 h after initial seeding to allow for sufficient cell adhesion. The duration of culture was adjusted for each cell type to reach confluency, i.e., SCs were given 48 h to attach and allow time to elongate [40], SNs require 72 h for sufficient neurite extension [36], and cocultures were cultured longer for additional cell integration. Cell alignment, elongation, and vertical neurite perforation into scaffolds were compared among the two groups.

### 2.7. Immunofluorescence Staining and Confocal Microscopy

Rhodamine phalloidin (Invitrogen) or fluorescein phalloidin (for SC-SN cocultures) (Invitrogen), and DAPI (4′,6′—Deiamidino-2-Phenylindole Dihydrochloride) (Abcam) were used to label cell cytoskeletal F-actin and cell nuclei, respectively. Beta-III tubulin monoclonal antibody (Invitrogen, 2G10, Waltham, MA, USA) was used in cocultures as a marker for SNs. Staining protocols were followed as recommended by the manufacturer. Briefly, PVDF-TrFE scaffolds were rinsed three times with PBS before fixing in 3.7% formaldehyde for 15 min at room temperature. Scaffolds were rinsed again thrice and permeabilized with 0.1% Triton X-100 at 4 °C for 5 min followed by additional PBS rinses. Scaffolds were then incubated in either fluorescein phalloidin for cocultures or rhodamine phalloidin at a dilution of 1:100 (10 µg/mL) in 2% BSA at 37 °C for 30 min followed by three PBS rinses. Alexa Fluor 488 goat anti-mouse IgG (Invitrogen) was diluted at 1:600 and added to the staining solution with rhodamine phalloidin before applying onto the samples. For SC-SN cocultures only, a second incubation with beta-III tubulin for 30 min followed after three PBS rinses. Alexa Fluor 594 goat anti-mouse IgG (Invitrogen) was diluted at 1:600 and added to the staining solution with fluorescein phalloidin (for cocultures). Mounting medium with DAPI (Abcam, Cambridge, UK) was added to the scaffolds which were then mounted on microscope slides, dried overnight, and sealed with clear nail polish.

Confocal microscopy was performed at Cincinnati Children’s Hospital confocal imaging core (CIC) on an inverted Nikon A1R confocal microscope. Maximum intensity profile (max IP) images were taken for determining the cell alignment and elongation, and z-stack images were taken for analyzing the neurite perforation into the scaffolds using Nikon Elements software.

### 2.8. Statistical Analysis

Values are reported as mean ± one standard deviation. Statistical and image analyses were performed in Microsoft Excel, ImageJ, GraphPad Prism 9.5.1, and NIS-Elements. One-way analysis of variance (ANOVA) with Tukey’s post hoc tests and Student *t*-tests were performed for stress-strain (Young’s moduli, ultimate tensile strength (UTS)) data of 1, 2, and 3 h aligned and unaligned PVDF-TrFE scaffolds to determine the statistical significance between groups (*n* = 5). Error bars indicate ± one standard deviation from data means. Scaffold porosity calculations (*n* = 5) were performed and analyzed similarly. Proliferation assays were repeated *n* = 3 times. One-way ANOVA with Tukey’s post hoc were performed for each group stimulated (or not) with ultrasound. *n* = 5 scaffolds each for SCs, SNs, and SC-SN cocultures for determining cell alignment, elongation, and perforation depth. At least 10 images were taken per slide, with at least 10 measurements per image. Cell alignment was determined via Fast Fourier Transform (FFT) alignment quantification in ImageJ. Cell elongation was calculated via aspect ratio measurement (for SCs) and neurite length measurement (for SNs), excluding neurites < 20 μm. Perforation depth measurements were completed in NIS-Elements via z-stack quantification. One-way ANOVA with Tukey’s post hoc tests were applied to all groups to determine statistically significant differences in perforation depth among conditions. A *p*-value ≤ 0.05 was considered significant. Statistical significance is presented as * *p* ≤ 0.05, ** *p* ≤ 0.005, and *** *p* ≤ 0.0005.

## 3. Results and Discussion

### 3.1. PVDF-TrFE Scaffold Physical Characterization

**Figure 2:**
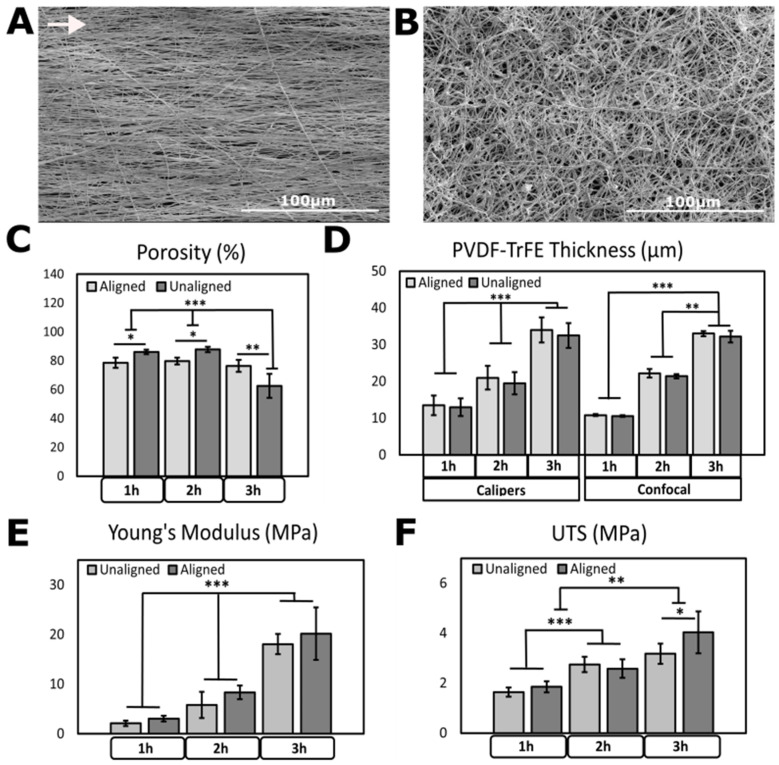
Mechanical testing of PVDF-TrFE scaffolds. (**A**) SEM image of aligned and (**B**) unaligned PVDF-TrFE scaffolds spun for 2 h at 500× magnification showing alignment. Arrow indicates direction of alignment. Scale bar = 100 µm. (**C**) Porosity (%) measurements. (**D**) Thickness measured for aligned and unaligned fibers, via calipers and confocal imaging, *n* = 5 unique scaffolds per condition. (**E**) Young’s moduli and (**F**) UTS of aligned and unaligned PVDF-TrFE, tested without directionality for unaligned and longitudinally (in direction of alignment) for aligned fibers, *n* = 5 scaffolds for each condition. All data are recorded for 1, 2, and 3 h scaffolds and reported as mean ± SD. * *p* ≤ 0.05; ** *p* ≤ 0.005; *** *p* ≤ 0.0005.

A total of 2 h aligned and 2 h unaligned PVDF-TrFE scaffolds were fabricated using electrospinning polymer solutions for 2000 RPM or 200 RPM, respectively. To demonstrate the feasibility of PVDF-TrFE for modeling engineered skin tissue, we examined scaffold thickness, porosity, Young’s modulus, and ultimate tensile strength. Previously, our lab has characterized the physical parameters of PVDF-TrFE, including fiber alignment, nanofiber diameter, and surface hydrophilicity [21] as well as quantified its piezoelectric capacity for use in nerve regeneration [33]. Figure 2A,B show SEM images of 2 h spun aligned and unaligned scaffolds taken at 500× magnification showing fiber arrangement. Additional SEM fiber characterization is available in our recently published work [34].

Scaffold porosity measurements (Figure 2C) show that all conditions produced porosity values above 75%, except 3 h spun unaligned scaffolds which had a 62.6% porosity. These values agree with previous data reported by Orkwis et al. but exceed other reported values (54%) [41], possibly due to using a highly evaporative solvent. Aligned scaffolds were less porous than unaligned scaffolds, as expected. However, longer spin times produced thicker scaffolds but did not significantly affect porosity in aligned scaffolds. Rather, unaligned scaffolds appear to be less porous when thickness was increased during longer spin times. Both aligned and unaligned scaffolds spun for 2 h are more porous compared to 1 and 3 h spun scaffolds. Material porosity in TESSs is important to allow cells to migrate and extend past the surface to form 3D cultures [41,42]. Studies have shown that scaffolds with 60–90% porosity are favorable for cutaneous healing applications as they provide sufficient oxygen and nutrient exchange, space for cellular activities, and the production of new ECM [43,44]. Adequate porosity also allows biomaterials to have sufficient nutrient transfer, molecular adsorption due to increased surface area, and mechanical properties [45].

Scaffold thickness (Figure 2D) was measured manually via calipers and verified with confocal imaging. Thickness trends of measured and imaged scaffolds appear consistent throughout each electrospinning time, with aligned scaffolds exhibiting greater thicknesses under all conditions. Approximately 10 μm are added to the thickness of the fiber mat for each additional hour spun. Scaffold thickness and electrospinning parameters agree with 20 μm-thick PVDF-TrFE measured in the literature [46].

Figure 2E,F and Figure S1 show Young’s moduli and UTS for 1, 2, and 3 h spun aligned and unaligned scaffolds, respectively. We previously observed that 2 and 3 h spun aligned fibers display greater Young’s moduli measured parallel to the fiber alignment compared to unaligned fibers, and 1 h spun unaligned scaffolds were stronger than respective aligned scaffolds [21]. In the context of PNS repair within cutaneous tissue, we aim for biocompatible fibrous scaffolds that are thin, porous, and biocompatible, yet able to withstand in vivo tensile forces. Young’s moduli averages for 1, 2, and 3 h aligned PVDF-TrFE generated from stress–strain curves were determined as 3.064 ± 0.580 MPa, 8.362 ± 1.386 MPa, and 20.19 ± 5.295 MPa, respectively. Young’s moduli averages for 1, 2, and 3 h unaligned scaffolds are 2.113 ± 0.554 MPa, 5.814 ± 2.625 MPa, and 18.06 ± 2.037 MPa, respectively. These values are notably less than values found in the literature. Bae et al. reported dry-spun PVDF-TrFE (75/25) fibers with thickness 34–37 μm to have an average Young’s modulus of 270 MPa [47]. Lee et al. [24] reported 225.28 ± 53.63 MPa for as-spun, aligned PVDF-TrFE, and Persano et al. reported moduli of 168 ± 8 MPa [48]. Young’s moduli of collagen fibrils representing the tensile strength of the wound microenvironment in normal and expanded skin were reported as 35.2 ± 27.0 and 46.5 ± 19.4 MPa, respectively [49], still greater than our reported values. Manschot et al. reported Young’s moduli values of healthy skin range between 4.6 and 20 Mpa [50], and Blair et al. recorded moduli of only 1.27 to 1.96 MPa for abdominal skin in tension [51]. Similar work was performed by Adadi et al. and results also show that aligned PVDF-TrFE (75/25) scaffolds were slightly stiffer (27.9 MPa) than unaligned scaffolds (15.9 MPa) [52]. Others measured healthy human skin to have Young’s modulus of 98.97 ± 97 MPa with UTS of 27.2 ± 9.3 MPa under tension [53], for thicknesses varying between 0.3 and 3 mm [54]. Pawlaczyk et al. reported lower tensile values of moduli, ranging from 70 MPa (child) to 60 MPa (elderly), with UTS of 21 MPa and 17 MPa, respectively [55].

Measured UTS values were determined as 1.852 ± 0.447 MPa, 2.581 ± 0.731 MPa, and 4.037 ± 1.681 MPa for aligned and 1.641 ± 0.371 MPa, 2.744 ± 0.621 MPa, and 3.177 ± 0.824 MPa for unaligned 1, 2, and 3 h PVDF-TrFE, respectively. The literature reports UTS of electrospun PVDF-TrFE to be 44.19 MPa [56], 40 MPa [49], and 28.9 MPa [57]. However, Lafrance et al. reported maximum tensile strength of cultured, living human skin equivalents to be between 0.871 and 1.169 MPa [58], which agrees with our numbers found for PVDF-TrFE. In cutaneous wound healing, it is encouraging that our fiber mats achieved Young’s moduli between 2.113 and 20.19 MPa, similar to the moduli of human skin recorded by Manschot et al. (4.6–20 MPa) [50]. Nonetheless, the range of moduli from other testing methods such as suction or indentation can vary greatly depending on the probe size used [59]. Indentation tests typically vary between Pa and tens of kPa [60,61,62]; suction tests report numbers about hundreds of kPa [63,64,65]; torsion tests are on the order of MPa [19,66]; and tensile measurements fall around a hundred MPa [67,68,69]. Thus, the literature reports six orders of magnitude difference in moduli for skin [70]. In comparison, traditional TESSs have moduli ranging from 0.271 MPa to 0.5463 MPa [71,72,73] and UTS between 0.01 and 0.086 MPa [74,75,76,77], which is closer to the moduli and UTS values of our PVDF-TrFE. Considering thickness, porosity, and tensile material performance, we selected 2 h spun aligned and unaligned PVDF-TrFE fibers to further investigate applications of modeling a TESS component.

### 3.2. Cell Alignment Quantification on Aligned and Unaligned PVDF-TrFE

**Figure 3:**
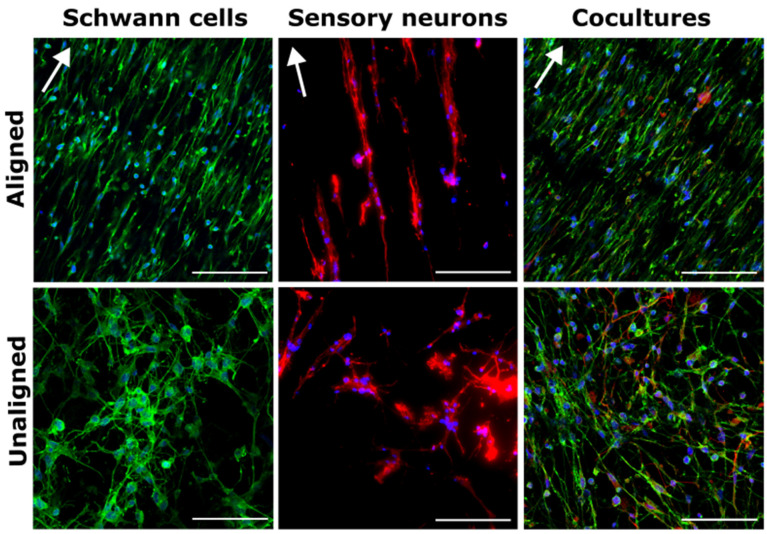
Aligned PVDF-TrFE promoted alignment of SCs, SNs, and SC-SN cocultures. Representative images of SCs, SNs, and SC-SN on aligned and unaligned PVDF-TrFE show cell alignment. Cytoskeleton labeled with fluorescein phalloidin (green) or rhodamine phalloidin (red), nuclei stained with DAPI (blue), and SNs labeled with β-III tubulin (red) in cocultures. Arrows indicate direction of fiber alignment. Scale bars = 100 μm.

**Figure 4:**
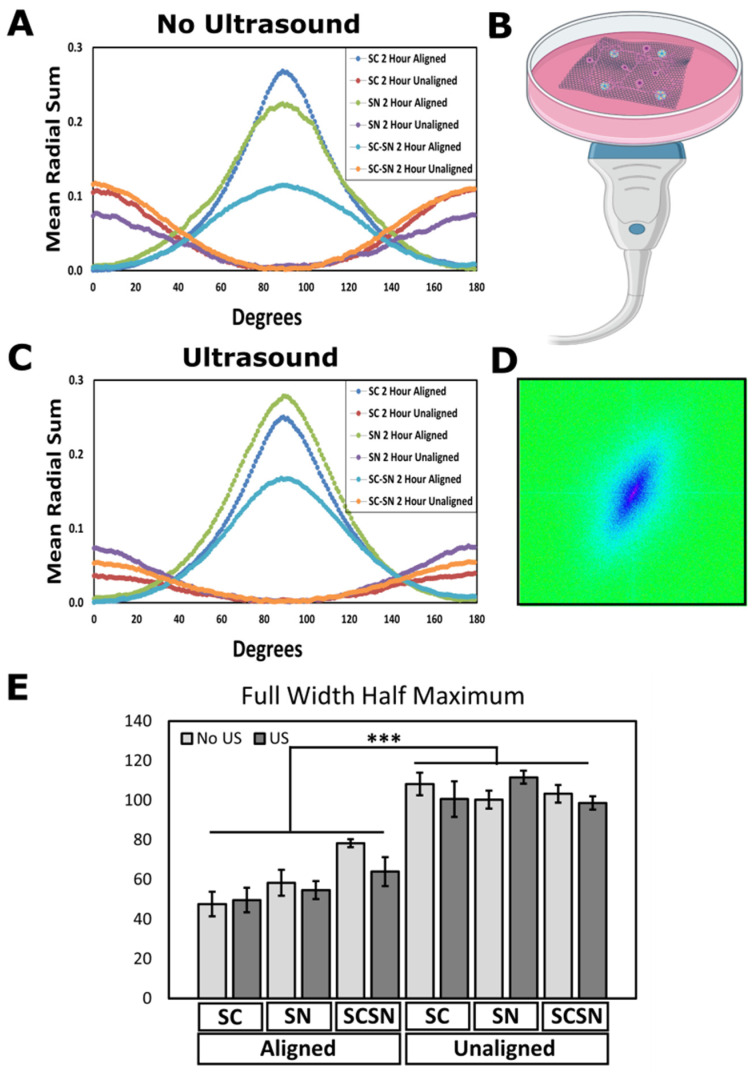
Ultrasound stimulation on aligned scaffolds promoted alignment of SCs and SNs. (**A**) Mean radial sums of control scaffolds (not treated with ultrasound) represent cell and neurite alignment. Mean radial sums curves generated from image oval profiles measured from 0° to 180°. (**B**) Schematic of ultrasound setup showing transducer and cell-seeded scaffold in dish. (**C**) Mean radial sums of scaffolds with ultrasound treatment. (**D**) Oval profile spectrum of FFT from immunofluorescence labeled images. (**E**) Full-width half-maximum values for SCs and SNs seeded separately and in coculture, on 2 h aligned and unaligned scaffolds, with and without ultrasound treatment. N = 3 unique scaffolds per condition with five images captured per slide, and LIPUS applied for 5 min for 3 days. Data reported as mean ± SD. *** *p* ≤ 0.0005.

To confirm the degree of alignment of SCs and SNs, we analyzed fluorescent microscopy images as previously performed by our lab [21,78]. Figure 3 shows the morphology of SCs, SNs, and SC-SN cultures on aligned and unaligned scaffolds. Full-width half-maximum values calculated from respective radial sums curves of Fast Fourier Transforms of the images were used for quantification to determine the extent of alignment of cell cytoskeletons. Figure 4 shows that aligned scaffolds for stimulated and unstimulated groups hold a high degree of cellular alignment by promoting alignment of SCs, SNs, and cocultures.

Activating PVDF-TrFE via ultrasound waves induces a piezoelectric response within the scaffold by creating mechanical deformations in the material. This produces an electrical response within the fibers. Ultrasound stimulation further enhanced this effect as seen in Figure 4C when compared to Figure 4A; although, full-width half maximum differences between stimulated and unstimulated groups are not statistically significant for either aligned or unaligned scaffolds. Interestingly, ultrasound stimulation even enhanced cellular alignment on most unaligned scaffolds compared to unstimulated, unaligned scaffolds. Aligned fibrous matrices are suitable for neuronal outgrowth and regeneration because the arrangement of fibers provides axon guidance cues to neuronal cells, a key aspect for neurogenic differentiation [79,80,81]. For instance, Jin et al. reported increased neurite outgrowth through use of an aligned fibrous structure around neuronal cells [82].

When considering PNS injury repair, cell alignment typically indicates a pro-regenerative phenotype as cells align along nerve wound gaps to form the basis for Bands of Büngner, the longitudinally aligned SC- and extracellular matrix-rich strands which guide regrowing axons [21,83,84]. Figure 4E shows cells were significantly more aligned on aligned scaffolds when compared to unaligned scaffolds, with no significant differences in alignment between cell types. Interestingly, however, ultrasound treatment slightly reduced alignment in SCs on aligned and SNs on unaligned scaffolds, although differences when compared to control scaffolds are not statistically significant. We predict that this behavior is partially attributed to ultrasound stimulation causing cells and neurites to migrate deep into scaffolds as opposed to aligning and elongating across the fibers. Despite variability in cell alignment for each scaffold type and treatment condition, both SCs and SNs appear to respond with alignment, which was strengthened in most cases when ultrasound was applied.

Previous work has shown PVDF-TrFE fibers promote adhesion and alignment in both SCs and fibroblasts [25,85,86]. Orkwis et al. showed that regardless of scaffold spin time (1, 2, or 3 h), aligned PVDF-TrFE resulted in SC and fibroblast alignment relative to cells on unaligned fibers [21]. Furthermore, Wu et al. demonstrated that aligned fibers support DRG growth and neurite extension, with maximal outgrowth seen in cocultures with SCs [85]. Similarly, Lee et al. showed more brainstem axons regenerated across aligned PVDF-TrFE spinal cord transplants when SCs were present [22,24], and Gryshkov et al. expressed the biocompatibility of PVDF-TrFE with SCs and its guiding properties for sensory neurite outgrowth [23], further supporting that PVDF-TrFE holding SCs are promising for neuronal repair. Moving forward, ultrasound stimulation of PNS cells on piezoelectric scaffolds may give rise to enhanced cell integration and vertical neurite outreach, which is highly desired in TESS applications. We hereby further investigate the SC and SN extension across and integration into PVDF-TrFE with ultrasound stimulation.

### 3.3. Verification of hK Viability on PVDF-TrFE

**Figure 5:**
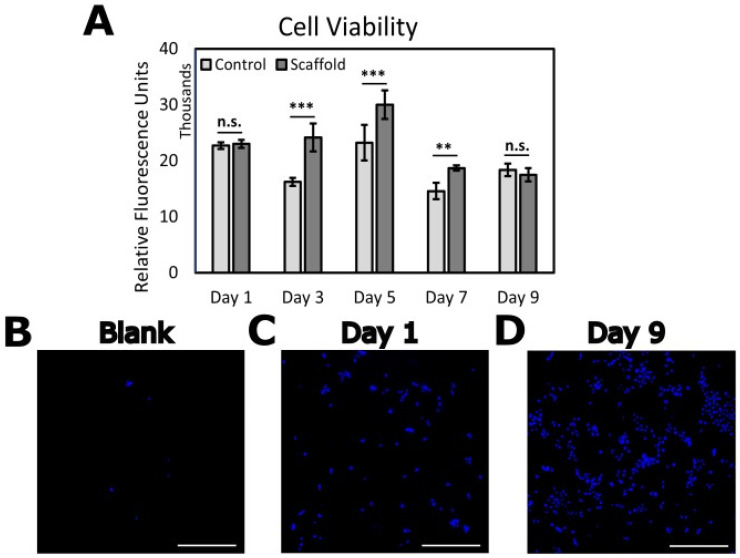
Viability of hKs seeded on 2 h aligned PVDF-TrFE scaffolds during the 9-day culture period. Cells seeded on Day 0 of assay. (**A**) Cell viability on PVDF-TrFE displayed via relative fluorescence units of Presto-blue reagent. hKs seeded at 10 k cells/well in a 12-well plate (*n* = 3). Controls are hKs seeded in polystyrene well plates with no PVDF-TrFE. Immunofluorescence images showing (**B**) PVDF-TrFE scaffold with no cells, (**C**) hKs on scaffold at Day 1, and (**D**) hKs on scaffold at Day 9 of assay. Scale bars = 500 μm. Cell nuclei stained with DAPI. All data reported as mean ± SD. ** *p* ≤ 0.005; *** *p* ≤ 0.0005. n.s.: no significance.

The epidermis layer of the skin consists of stratified squamous epithelium. To mimic the behavior of a functional epidermal layer in full-thickness skin grafts, it is necessary to grow and differentiate keratinocytes, or in some cases epidermal progenitor cells, into a thin sheet to be transplanted onto a dermal substrate [87]. We hereby verified the viability via proliferation of hKs on 2 h aligned PVDF-TrFE over 9 days of culture. Figure 5A shows cell viability via a measure of relative fluorescence units (RFU) across three unique samples, seeded at 10k hKs per scaffold, compared to controls with no PVDF-TrFE (blank) (Figure S2). Immunofluorescence images (Figure 5B–D) demonstrate sufficient hK proliferation on PVDF-TrFE from Day 1 to Day 9 (5B indicates PVDF-TrFE with no cells).

Keratinocytes proliferate in the basal layer and migrate through suprabasal cell layers as they begin to differentiate, migrating toward the skin’s surface where they are eventually shed. Achieving equilibrium between the proliferation and differentiation stages is crucial for maintaining homeostasis and a healthy barrier function [88]. First, however, it is important to verify cell viability on a new substrate which allows for the processes of proliferation and differentiation. Cell adhesion and viability is predominantly influenced by the physical and chemical characteristics of the material’s surface. Surface properties such as topography [89], wettability [90], and mechanics [91] can determine the regenerative cellular responses including migration, proliferation, and differentiation [21,92,93]. Thus, recognizing these cell-scaffold interactions is critical for engineering advanced biomaterials for use in skin wound healing. Compared to our findings, Teixeira et al. observed a similar trend in proliferation after a 7-day culture of hKs on PVDF-TrFE/barium titanate [94]. Further, Ranjbarvan et al. performed an MTT assay to quantify the viability of neonate keratinocytes on electrospun polycaprolactone-platelet gel scaffolds for a 10-day culture period, which also agrees with our findings [95]. hK viability on PVDF-TrFE allows for future work involving the differentiation of hKs to render a full-thickness, stratified epidermal layer.

### 3.4. Ultrasonic Stimulation of Cells on PVDF-TrFE

Here, we activated PVDF-TrFE via non-invasive LIPUS to induce a piezoelectric response within the scaffold. From the electrical response caused by the mechanical deformations of ultrasonic waves, the behavior of stimulated SCs and SNs on scaffolds can be explored. For this work, we analyzed cell elongation and neurite extension on aligned and unaligned scaffolds as well as perforation depth for future potential to serve as an innervated, in vitro skin model Figure S3. We chose LIPUS for its safe, non-invasive, and localized application. Applying LIPUS to PVDF-TrFE has potential for addressing the lack of nerve integration in TESSs through the investigation of neurite extension and perforation into the scaffold. It was observed that ultrasound stimulation promoted SC elongation and SN extension on aligned PVDF-TrFE, while an opposite trend is observed in unaligned scaffolds, indicating that SCs and SNs do not extend along fibers, but rather, we believe cells extend downward into the scaffold.

**Figure 6:**
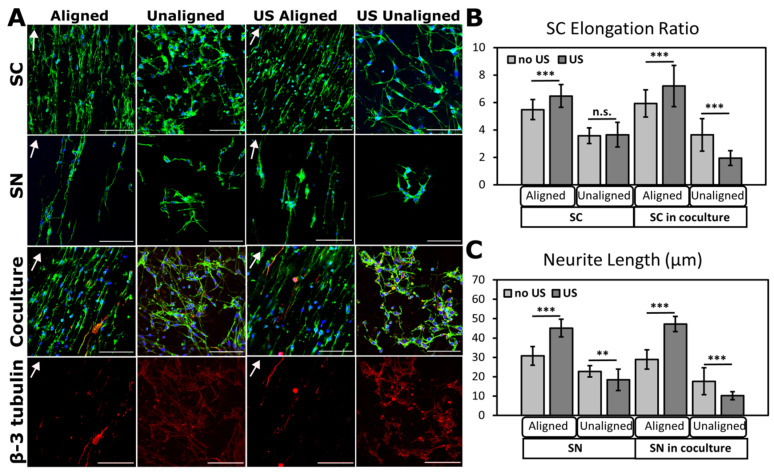
Ultrasound stimulation of SCs and SNs seeded on PVDF-TrFE scaffolds electrospun for 2 h promoted SC elongation and neurite extension in aligned scaffolds. (**A**) Representative confocal microscopy images of SCs and SNs seeded separately and in coculture on aligned and unaligned PVDF-TrFE scaffolds stimulated with ultrasound. Cytoskeleton stained with fluorescein phalloidin, nuclei stained with DAPI, and SNs labeled with beta-III tubulin in cocultures. Beta-III tubulin-only images show neurites in cocultures. Arrows indicate direction of fiber alignment. Scale bars = 100 μm. (**B**) SC elongation and (**C**) neurite extension on aligned and unaligned scaffolds with and without ultrasound treatment. Culture times were 48 h for SCs, 72 h for SNs, and 5 days for SC-SN cocultures for stimulated and unstimulated scaffolds. Data reported as mean ± SD. ** *p* ≤ 0.005; *** *p* ≤ 0.0005. n.s.: no significance.

Figure 6A shows immunofluorescent images of SCs and SNs seeded individually and in coculture, with and without ultrasound stimulation. SC elongation (Figure 6B) calculated via aspect ratio and SN extension (Figure 6C) demonstrate that cells cocultured on aligned scaffolds with applied ultrasound have the greatest cell elongation and neurite extension. Ultrasound on unaligned scaffolds caused SCs and SNs to perforate deep into scaffolds as opposed to elongating or extending across fibers, hence a decrease in SC elongation and SN extension was observed.

**Figure 7:**
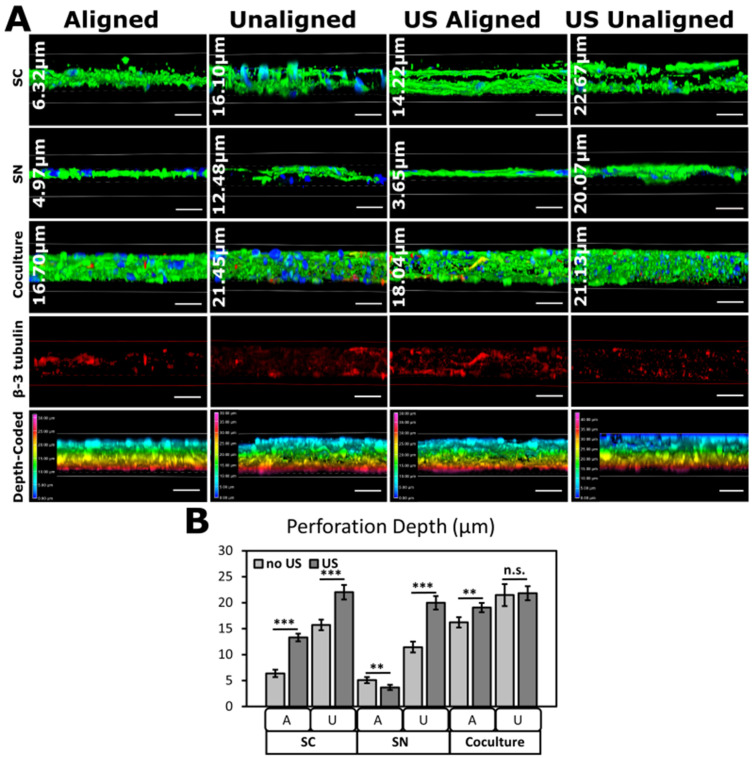
Perforation depth of SCs and SNs vertically into PVDF-TrFE. Ultrasound promoted perforation of SCs and SNs on unaligned scaffolds. (**A**) Representative immunofluorescence images of SCs and SNs seeded individually and in coculture on aligned and unaligned scaffolds with or without ultrasound treatment. Average perforation depth (µm) representative of a specific sample displayed on each image. Beta-III tubulin-only images show perforation of neurites in cocultures. Color-coded alpha-blended projections show perforation depth from XZ plane. Scale bars = 20 μm. (**B**) Quantified perforation depth of SCs and neurites with and without ultrasound. Culture times were 48 h for SCs, 72 h for SNs, and 5 days for SC-SN cocultures for stimulated and unstimulated scaffolds. Data reported as mean ± SD. ** *p* ≤ 0.005; *** *p* ≤ 0.0005. n.s.: no significance.

Cytoskeletal and neurite perforation depth throughout aligned and unaligned scaffolds, with and without ultrasound stimulation show that ultrasound induced greater cell perforation in most scaffolds (Figure 7). Cellular perforation depth into scaffolds shows that maximum perforation of cytoskeletons and neurites is achieved in unaligned scaffolds with ultrasound stimulation (Figure 7B). SNs on aligned PVDF-TrFE, however, did not seem to extend into the scaffold after stimulation, but rather, elongate across the fibers as supported by results in Figure 6. No statistical significance was observed between ultrasound and no ultrasound on cocultures seeded on unaligned PVDF-TrFE, likely because cells perforated the full depth of the scaffold. The 2 h unaligned scaffolds are approximately 21 ± 0.47 μm in depth (measured via confocal imaging), and because of this, we are certain that cells have reached the maximum scaffold depth.

Figure 6 and Figure 7 show that applying LIPUS to SCs and SNs enhances cytoskeletal and neurite outreach. Cells and neurites elongate when seeded on aligned scaffolds, whereas they more easily perforate through unaligned scaffolds. Ultrasound stimulation appears to enhance these processes by magnifying SC aspect ratio and neurite extension. For TESS applications, it is desired that skin substitutes exhibit maximal vertical neurite extension such that SNs extend throughout the full depth of the dermis. In cocultures in Figure 7, SNs are located near the top and bottom of the scaffold, suggesting that SNs follow SCs deeper into the scaffold. Interestingly in SCs, ultrasound appears to cause a separation in cell layers on both aligned and unaligned scaffolds as seen in Figure 7A, which may be due to the induced piezoelectric response in PVDF-TrFE.

In vitro studies show that ultrasound stimulation of dorsal root ganglia neurons increased neurite outgrowth by two-fold compared to unstimulated controls [96,97], which supports our findings. Further, Ito et al. found that ultrasound encouraged axonal outgrowth through decreasing GSK-3β and axonal semaphorin 3A expression, which is an inhibitor of axonal regeneration and a potential inhibitor of axonal regrowth, respectively [29]. Recent studies also suggest ultrasound stimulation can modulate diverse pathways in SCs through varying ECM composition and interactions between the ECM and its receptors [98,99], possibly explaining the separation in SC layers on stimulated scaffolds. Vertical neurite outgrowth throughout TESSs is crucial for proper neuronal development, formation of nerve networks, and accelerated healing [7].

## 4. Conclusions

In this work, we investigated electrospun PVDF-TrFE to create an innervated and mechanically strong substrate for use as a tissue-engineered skin scaffold. A non-invasive LIPUS modality was utilized to activate the PVDF-TrFE scaffold to further enhance SC elongation and SN extension for inducing regenerative cell phenotypes. Scaffold thickness, porosity, and tensile tests showed that 2 h electrospun aligned and unaligned PVDF-TrFE fiber mats were optimal for this application. Cell and neurite alignment on aligned fibers was verified via analysis of FFT quantification, and the results agree with previous studies that SCs and SNs appear to respond positively with alignment. This response was strengthened in scaffolds treated with ultrasound stimulation. Additionally, we evaluated PVDF-TrFE for epidermal substitutes by investigating hK viability to verify scaffold compatibility for future hK differentiation into a fully stratified epidermal layer. Finally, we further explored the cell response of elongation, neurite extension, and perforation when scaffolds are subjected to ultrasound. Our results show enhanced SC elongation and neurite extension on aligned scaffolds and increased perforation depth of cells on unaligned scaffolds. Ultimately, these data are valuable in selecting PVDF-TrFE parameters appropriate for constructing an innervated, in vitro, cutaneous model.

## Data Availability

The raw data for the above work was produced at the University of Cincinnati. Acquired data supporting the findings of this study are available upon reasonable request from the authors.

## Supplementary Materials

The following are available online at https://www.mdpi.com/2313-7673/9/1/2/s1, Figure S1: Representative stress-strain curves from one unique aligned and unaligned PVDF-TrFE sample scaffold of (A) 1-, (B) 2-, and (C) 3-hour fabrication times. Figure S2: Presto-Blue calibration curve generated from relative fluorescence units of human keratinocytes seeded at densities ranging from 0 to 50,000 cells/well. Figure S3: Representative immunofluorescence images showing cell measurements taken. (A) Schwann cell length and width measurements for aspect ratio calculations and (B) neurite length measured from center of the nearest nuclei. Neurites < 20 μm excluded.
