# Supplementary material for: Innervation of an Ultrasound-Mediated PVDF-TrFE Scaffold for Skin-Tissue Engineering
Source: Biomimetics (Basel). 2023 Dec 20;9(1):2. doi: 10.3390/biomimetics9010002 (PMC11154284; doi:10.3390/biomimetics9010002)
Supplement: Supplementary file 1 [file biomimetics-09-00002-s001.zip › biomimetics-2735812-supplymentary.pdf]

Innervation of an Ultrasound-Mediated PVDF-TrFE Scaffold for Skin-Tissue Engineering

Jennifer A. Westphal [1], Andrew E. Bryan [2], Maksym Krutko [1], Leyla Esfandiari [1,3,4], Stacey C. Schutte [1] and Greg M. Harris [1,2,5,*]

[1] Department of Biomedical Engineering, University of Cincinnati, Cincinnati, OH 45221, USA

[2] Department of Chemical and Environmental Engineering, University of Cincinnati, Cincinnati, OH 45221, USA

[3] Department of Environmental and Public Health Sciences, University of Cincinnati, Cincinnati, OH 45267, USA

[4] Department of Electrical and Computer Science, University of Cincinnati, OH 45221, USA

[5] Neuroscience Graduate Program, University of Cincinnati College of Medicine, Cincinnati, OH 45221, USA

* Correspondence: gregory.harris@uc.edu; Tel.: +1-(513)-556-4167; Fax: (513)-556-3473

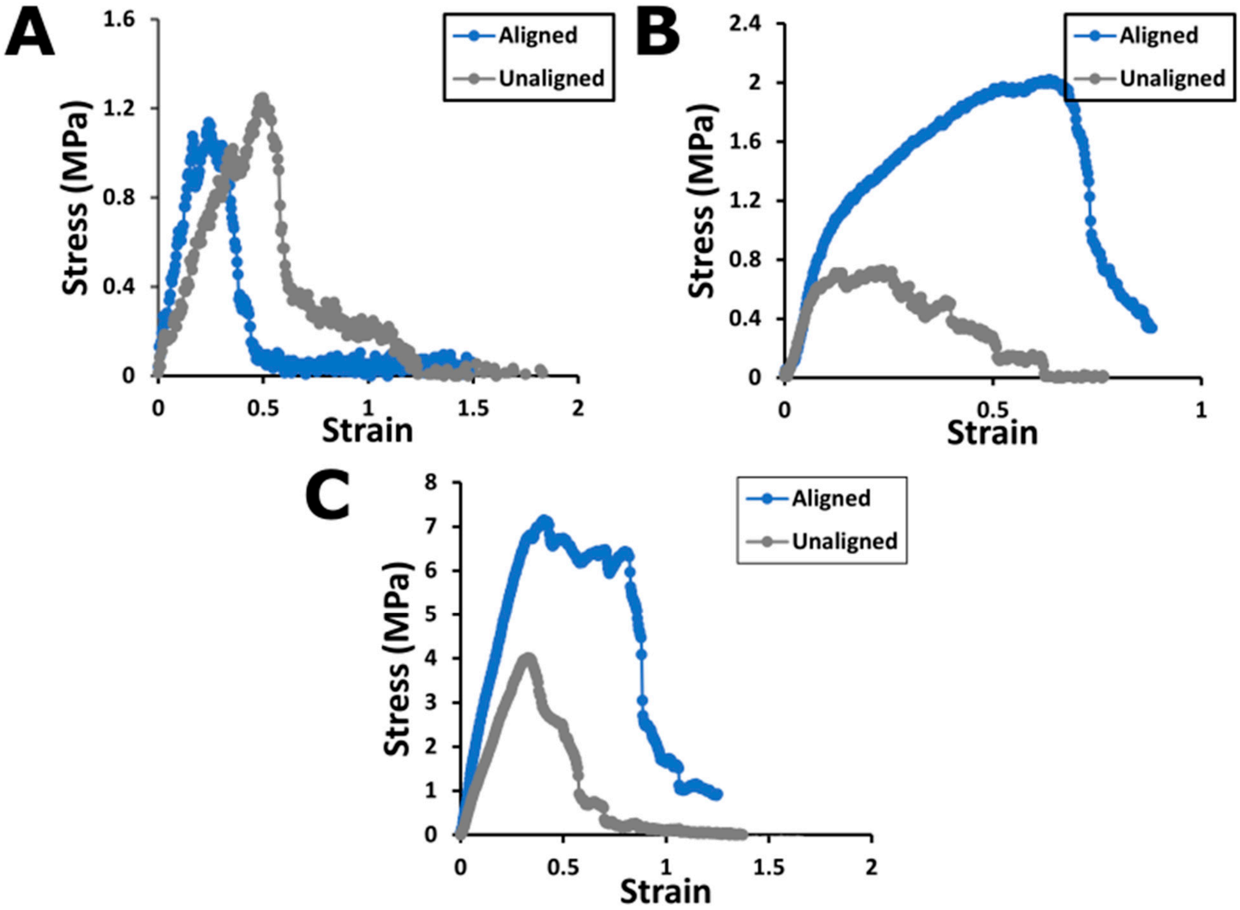

Figure S1. Representative stress-strain curves from one unique aligned and unaligned PVDF-TrFE sample scaffold of A) 1-, B) 2-, and C) 3-hour fabrication times.

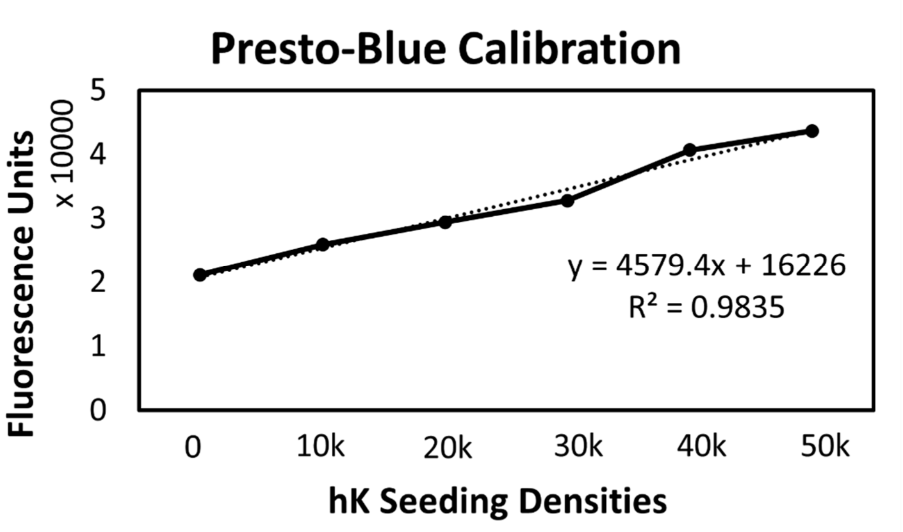

Figure S2. Presto-Blue calibration curve generated from relative fluorescence units of human keratinocytes seeded at densities ranging from 0 to 50,000 cells/well.

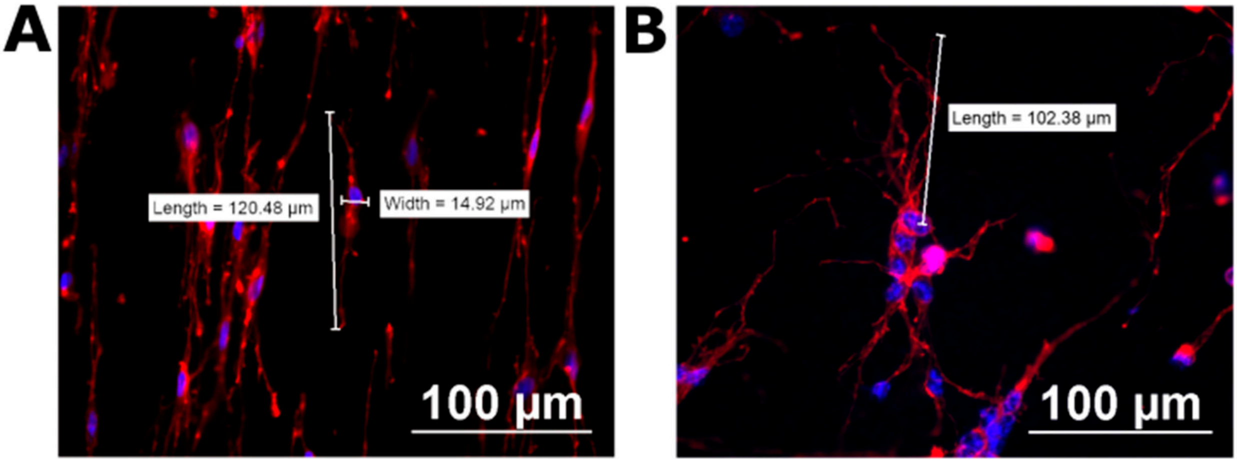

Figure S3. Representative immunofluorescence images showing cell measurements taken. A) Schwann cell length and width measurements for aspect ratio calculations and B) neurite length measured from center of the nearest nuclei. Neurites <20 µm excluded.
